# Characteristics of the specific humoral response in patients with advanced solid tumors after active immunotherapy with a VEGF vaccine, at different antigen doses and using two distinct adjuvants

**DOI:** 10.1186/s12865-017-0222-z

**Published:** 2017-07-26

**Authors:** Javier Sánchez Ramírez, Yanelys Morera Díaz, Mónica Bequet-Romero, Francisco Hernández-Bernal, Katty-Hind Selman-Housein Bernal, Ana de la Torre Santos, Eduardo Rafael Santiesteban Álvarez, Yenima Martín Bauta, Cimara H. Bermúdez Badell, Josué de la Torre Pupo, Jorge V. Gavilondo, CENTAURO-2 Team of Investigators, Marta Ayala Avila

**Affiliations:** 1 Department of Pharmaceuticals, Center for Genetic Engineering and Biotechnology (CIGB), P.O. Box 6162, Playa Cubanacán, Havana, 10600 Cuba; 2 Department of Clinical Research, CIGB, P.O. Box 6162, Playa Cubanacán, Havana, 10600 Cuba; 3 Center of Medical and Surgical Research (CIMEQ), Playa, Siboney, Havana, 12100 Cuba; 4 “Celestino Hernández Robau” Hospital, Santa Clara, 50100 Cuba; 5 “José Ramón López Tabranes” Hospital, Matanzas, 40100 Cuba

**Keywords:** CIGB-247, VEGF, Cancer vaccine, Humoral response, Clinical trial

## Abstract

**Background:**

CIGB-247, a VSSP-adjuvanted VEGF-based vaccine, was evaluated in a phase I clinical trial in patients with advanced solid tumors (CENTAURO). Vaccination with the maximum dose of antigen showed an excellent safety profile, exhibited the highest immunogenicity and was the only one showing a reduction on platelet VEGF bioavailability. However, this antigen dose level did not achieve a complete seroconversion rate in vaccinated patients. These clinical results led us to the question whether a “reserve” of untapped immune response potential against VEGF could exist in cancer patients. To address this matter, CENTAURO-2 clinical trial was conducted where antigen and VSSP dose scale up were studied, and also incorporated the exploration of aluminum phosphate as adjuvant. These changes were made with the aim to increase immune response against VEGF.

**Results:**

The present study reports the characterization of the humoral response elicited by CIGB-247 from the combining of different antigen doses and adjuvants. Cancer patients were immunologically monitored for approximately 1 year. Vaccination with different CIGB-247 formulations exhibited a very positive safety profile. Cancer patients developed IgM, IgG or IgA antibodies specific to VEGF. Elicited polyclonal antibodies had the ability to block the interaction between VEGF and its receptors, VEGFR1 and VEGFR2. The highest humoral response was detected in patients immunized with 800 μg of antigen + 200 μg of VSSP. Off-protocol long-term vaccination did not produce negative changes in humoral response.

**Conclusions:**

Vaccination with a human VEGF variant molecule as antigen in combination with VSSP or aluminum phosphate is immunogenic. The results of this study could contribute to the investigation of this vaccine therapy in an adequately powered efficacy trial.

**Trial registration:**

Trial registration number: RPCEC00000155. Cuban Public Clinical Trial Registry. Date of registration: June 06, 2013. Available from: http://registroclinico.sld.cu/.

**Electronic supplementary material:**

The online version of this article (doi:10.1186/s12865-017-0222-z) contains supplementary material, which is available to authorized users.

## Background

Over the last decade promising results on cancer vaccines have been achieved in clinical trials and the field is rapidly expanding [1]. An attractive approach is the development of vaccines against molecular markers expressed in the tumor vasculature or directly against one of the most prominent molecular angiogenic players: the vascular endothelial growth factor (VEGF). This type of strategy, known as specific active immunotherapy, has been mostly developed in the oncological arena. VEGF is one of the most important growth factors with a relevant role on tumor angiogenesis, and it has become an attractive target for cancer immunotherapy [2]. Within this research line, our group has developed CIGB-247, a VEGF-based vaccine, that uses a recombinant human VEGF variant molecule as antigen [3] in combination with VSSP, a bacterial-derived adjuvant [4].

CIGB-247 has previously shown anti-tumor and anti-metastatic effects in mice, stimulating the development of VEGF-blocking antibodies and specific T cell responses [3, 5, 6]. After extensive preclinical studies [5, 7], this vaccine candidate was evaluated between 2011 and 2012 in a Phase I clinical trial (code name CENTAURO), where safety, tolerance, and immunogenicity were studied in 30 patients with advanced solid tumors [8].

The CENTAURO study was a first-in-human phase 1 trial to evaluate a cancer therapeutic vaccine based on human VEGF. This clinical trial included three antigen levels (50, 100 and 400 μg), all in combination with 200 μg of VSSP, delivered subcutaneously once a week for 8 weeks, with a booster re-immunization on week 12. CIGB-247 showed an excellent safety and tolerance profile, and was immunogenic at the three studied antigen doses. Results suggested an antigen dose effect on immunogenicity. The immunogenicity increased with higher antigen doses, in terms of the number of patients with anti-VEGF IgG antibodies, the ability of serum to block VEGF/VEGF receptor 2 (VEGFR2) interactions, and positivity in specific gamma-IFNγ ELISPOT tests. Patients with higher accumulated survival times were positive to all these immune response tests. The higher antigen dose patient cohort (400 μg of antigen + 200 μg of VSSP) was the only one showing a reduction on platelet VEGF bioavailability, indicating a functionality of induced antibodies. However, this group did not achieve a complete seroconversion rate [8].

Clinical results of the CENTAURO study led us to the question whether a “reserve” of untapped immune response potential against VEGF could exist in cancer patients, which could be further manipulated by increasing the amount of antigen. As experimental basis for this matter, our group achieved satisfactory results in non-human primates by increasing the amount of antigen in combination with VSSP as adjuvant and using a weekly vaccination scheme [7]. VSSP has shown immunopotentiating properties on the humoral and cellular responses [9–11], however, it has not yet been explored whether increasing the amount of VSSP in the vaccine lead to higher antibody titers specific to VEGF.

An alternative strategy to enhance the vaccine immunogenicity is changing adjuvant composition in the CIGB-247 vaccine formulation. Up to date, aluminum salts are considered as the gold standard because of their effectiveness at enhancing antibody responses and their strong safety records among human adjuvants [12]. Hence, we developed a variant of CIGB-247 that incorporates aluminum phosphate as adjuvant. VEGF antigen formulated in aluminum was found to be safe in two mouse strains and in non-human primates; in mice, it inhibits tumor growth and metastases, and elicits anti-VEGF blocking antibodies and cell-mediated direct cytotoxic responses [13]. However, it has not been tested whether VEGF formulated in aluminum produces or not higher specific IgG antibody titers and VEGF/VEGFR2 blocking activities with respect to the same antigen dose per injection and VSSP as adjuvant.

Based on the unanswered questions, we firstly tested in pre-clinical animal models all of the above mentioned changes in the vaccine formulation. Then, these changes were explored in a second phase I (b) clinical trial (code name CENTAURO-2) that involved CIGB-247 vaccine candidate. This clinical trial was done in patients with advanced solid tumors. Different antigen doses and adjuvants were evaluated in terms of safety and immunogenicity, taking as reference 400 μg of antigen + 200 μg of VSSP, previously evaluated in the CENTAURO study. Because of the importance of anti-VEGF antibodies in the vaccine’s potential anti-tumor effects, the present paper is mainly devoted to the description and discussion of the humoral response results of the trial, and of those obtained during the follow up of surviving patients that continued to be vaccinated off-trial.

## Methods

### Investigational product

The antigen used in this study is a recombinant fusion protein, representative of human VEGF isoform 121 [3]. The lyophilized antigen was produced under GMP conditions in vials of 400 μg (lots VED 12403/0 and VEN 13401/0) by the Development Unit of Center for Genetic Engineering and Biotechnology (CIGB, Havana, Cuba).

The adjuvants used were aluminum phosphate (lots VAN 1301/0 and VAN 1303/0) and VSSP (lot 711301). VSSP are very small sized particles obtained from the *Neisseria meningitides* outer membrane, supplied by the Center for Molecular Immunology of Havana, Cuba. Both adjuvants were produced under GMP conditions. At the moment of vaccination, one or two antigen vials were dissolved in pre-calculated amounts of injection water, and the required amount was mixed with the established quantity of VSSP or aluminum phosphate, up to a final volume never exceeding 0.25 mL (mice) or 1 mL (rabbits, non-human primates and patients) per injection dose.

### Animals

Female C57BL/6 and Balb/c mice, 7–9 weeks of age housed under pathogen-free conditions, were maintained at five animals per cage in contained areas. Female New Zealand rabbits weighting 1.5–2 kg (7–8 weeks of age) and healthy adult green monkeys (Chlorocebus-formerly Cercopithecus-aethiops sabaeus) weighting 3–7 kg, were caged individually in special tasked areas. All animals were purchased from the National Center for Animal Breeding (CENPALAB, Havana, Cuba), and maintained in the animal facility of the Center for Genetic Engineering and Biotechnology in accordance with the Cuban guidelines for the care and use of laboratory animals. All studies were approved by the Institute’s Animal Care and Use Committee.

### Pre-clinical study for the evaluation of VEGF-specific antibody response elicited at different VSSP doses and using two distinct adjuvants

Immunization was done subcutaneously in mice, rabbits and non-human primates. The immunization scheme with VSSP as adjuvant comprised eight weekly vaccinations, meanwhile using aluminum phosphate, the schedule included four bi-weekly administrations.

Humoral response was followed using an ELISA test for specific anti-human VEGF antibody titer and a competitive ELISA test for serum blockade on VEGF/VEGFR2 interaction, as previously described [3, 5, 7].

### Design of the Centauro-2 trial and immunization protocol

The CENTAURO-2 clinical trial was a phase Ib, multicenter, open, non-controlled study of the CIGB-247 cancer vaccine, where different antigen doses and adjuvants were evaluated in fifty patients with advanced solid tumors. Written informed consent was obtained for all patients. The protocol and patient informed consent forms were approved by the hospitals institutional review boards and ethics committees, and by the Cuban Regulatory Authority (CECMED). This study was conducted in accordance with the ethical guidelines of the Declaration of Helsinki.

Patients were enrolled by the CIMEQ (Havana, Cuba), Celestino Hernández Robau (Santa Clara, Cuba) and José Ramón López Tabranes (Matanzas, Cuba) hospitals. Inclusion and exclusion criteria were similar to those applied for CENTAURO study [8]. However, patients with brain metastases were excluded of this trial.

**Fig. 1:**
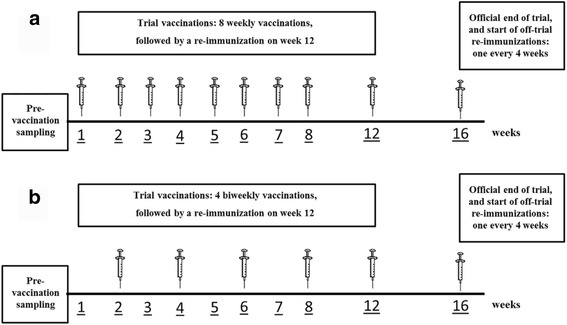
Vaccination schedules. CIGB-247 combinations using VSSP or aluminum phosphate as adjuvants were administered weekly (**a**) or bi-weekly (**b**), respectively. Pre-vaccination sampling included sera and plasma. After the end of the trial period (week 16), a re-immunization was done once every 4 weeks until death, intolerance, marked disease progression or patient’s withdrawal of consent

Fifty patients were randomly allocated in each of the five vaccination cohorts (10 individuals per group), corresponding to: (a) 400 μg of antigen + 200 μg of VSSP referred herein as Ag + V or reference group (maximum dose previously evaluated in CENTAURO study); (b) 400 μg of antigen + 400 μg of VSSP referred herein as Ag + 2 V; (c) 800 μg of antigen + 200 μg of VSSP referred herein as 2Ag + V; (d) 200 μg of antigen + 0.7 mg Al^3+^ referred herein as ½Ag + Al; (e) 400 μg of antigen + 0.7 mg Al^3+^ referred herein as Ag + Al. All vaccinations were administered subcutaneously as a single site dose. Figure 1a details the immunization protocol for groups Ag + V, Ag + 2 V and 2Ag + V, and Fig. 1b for the ½Ag + Al and Ag + Al cohorts.

At week sixteen, data from each patient were gathered and processed for the final report to be submitted to CECMED. Individuals surviving the trial period were eligible under medical supervision to start off-trial voluntary re-immunizations. Re-immunizations started on week sixteen, once every four weeks, until death, intolerance, marked disease progression or patient’s withdrawal of consent.

### Human blood samples

Venous blood samples were collected using a blood collection set with pre-attached holder (Becton Dickinson 367355) and taken into an EDTA tube or into a serum separator tube for plasma and serum analyses respectively. Serum and plasma samples were immediately stored at -70 °C until use.

Blood samples were taken during the trial period at weeks 0 (pre-vaccination), 5 or 6, 9, 13 (one week after the end of trial vaccinations) and 16 (end of trial period and start of off-trial re-immunizations). For investigations conducted during the off-trial re-immunizations, blood samples were taken at different time points, depending on patient availability.

### ELISAs reagents

GST-fused human VEGF isoform 121 (GST-hVEGF) was produced in *E. coli* as previously described [14]. Human VEGF isoform 121 (rhVEGF) was produced in CHO cells [15]. Skim milk powder (A0830) and Tween 20 (A1389) were supplied by AppliChem. HRP-conjugated sheep anti-mouse IgG antibody (Sigma A6782) or HRP-conjugated goat anti-rabbit IgG antibody (Sigma A0545) were used for detecting mouse or rabbit serum IgG respectively. HRP-conjugated goat anti-human IgG (Fc γ fragment specific) antibody (Jackson Immunoresearch Laboratories, 109-035-098) was used for detecting human or monkey serum IgG at 80 ng/mL. Biotinylated antibodies specific for human IgM (3840-6-250), human IgA (3860-6-250) and human IgE (3810-8-250) were supplied by Mabtech. Biotinylated mouse monoclonal antibodies specific for human IgG1 (ab9975), IgG2 (ab99785), IgG3 (ab99830) and IgG4 (ab99824) were purchased from Abcam. Recombinant human VEGF receptor 2/Fcγ chimera (Sigma, V6758) and recombinant human VEGF receptor 1/Fcγ chimera (Sigma, V1385) were used in competitive ELISAs as described below. Bevacizumab, a commercially available monoclonal antibody specific to human VEGF (Roche, Switzerland) was used as a positive control for VEGF/VEGFR2 and VEGF/VEGFR1 blockade. In the competitive ELISAs, biotinylated goat antibodies specific for human VEGFR2 (BAF357) or human VEGFR1 (BAF321) were supplied by R&D Systems for detecting VEGF/VEGFR2 or VEGF/VEGFR1 bindings respectively, and used at 0.1 μg/mL. Streptavidin-peroxidase conjugate (Sigma, S5512), was used at 1/30,000 dilution.

### ELISA for specific anti-human VEGF IgG, IgM, IgA and IgE antibodies

The levels of human IgG, IgM, IgA and IgE antibodies against rhVEGF were measured by a conventional isotype-specific indirect ELISA. Wells were coated with rhVEGF (2.5 μg/mL) during overnight incubation at 4 °C (100 μL/well). Following blocking step (250 μL/well), the wells were incubated with serum samples (100 μL/well, 1 h at 37 °C) and IgG, IgM, IgA or IgE antibodies were detected with HRP-conjugated goat anti-human IgG antibody, biotinylated goat anti-human IgM antibody (specific for Fc5μ), biotinylated anti-human IgA monoclonal antibody (specific for Fc part) and biotinylated anti-human IgE monoclonal antibodies respectively. For biotinylated conjugates the detection system consisted of a 1:30,000 dilution of streptavidin-conjugated HRP (100 μL/well, 45 min at 37 °C). Plates were developed by using H_2_O_2_ as substrate and OPD or TMB as chromogen (100 μL/well). After 15 min, the reaction was stopped by the addition of 2.0 N H_2_SO_4_ (50 μl/well), and the absorbance was measured at 492 or 450 nm, respectively.

For IgG assay, the wells were blocked with 2.5% goat serum, 2% skim milk, 0.05% Tween20. Serum samples were diluted with blocking buffer. Secondary antibody was diluted with 2% skim milk, 0.05% Tween20. IgG anti-VEGF ELISA has been previously described in details by Sánchez et al. [15]. ELISAs for detecting IgM, IgA and IgE antibodies specific to VEGF used as blocking reagent the following buffer: 2.5% goat serum, 2% BSA, 0.05% Tween20. Serum samples were diluted with RD6 (R&D Systems, diluent of kit SVE00). Biotinylated conjugates and streptavidin-conjugated HRP were diluted in 1% BSA.

IgG antibody titer was estimated as previously described [15]. The procedure was similar for IgM, IgA and IgE with the difference that the interpolated value on “x” axis was determined by adding five standard deviations to the duplicated mean of the blank optical density.

Titer ratio and “VEGF-specific antibody titer” were calculated as follow:

$$ \mathrm{Titer}\ \mathrm{ratio}=\frac{\mathrm{Post}\ \mathrm{vaccination}\ \mathrm{titer}}{ \Pr \mathrm{e}\ \mathrm{vaccination}\ \mathrm{titer}}\ (A) $$

$$ \mathrm{Specific}\ \mathrm{antibody}\ \mathrm{titer} = \mathrm{P}\mathrm{ost}\ \mathrm{vaccination}\ \mathrm{titer}\hbox{-} \mathrm{P}\mathrm{r}\mathrm{e}\ \mathrm{vaccination}\ \mathrm{titer}\ \left(\mathrm{B}\right) $$

To declare a given serum sample taken during vaccination to be positive for VEGF-specific IgG, IgM, IgA, or IgE antibodies, the obtained “titer ratio” must be ≥2 (formula A). In the particular case of IgG antibodies, additionally to the criterion depicted above, for a sample to be considered positive, it has also to comply with a value of “specific antibody titer” ≥1/100 (formula B).

The term seroconversion is only used in this paper for IgG antibodies and refers to a patient that has shown two or more samples positive for VEGF-specific antibodies during trial vaccinations or off-trial re-immunizations (seroconverted patient) [8].

### IgG subclasses assays

Antigen-specific IgG1, IgG2, IgG3, and IgG4 antibodies were determined by indirect ELISA using biotinylated mouse monoclonal anti-human subclass-specific antibodies (IgG1, IgG2, IgG3 and IgG4). Briefly, microtiter plates were coated with rhVEGF (2.5 μg/mL) and blocked with 4% BSA. Sera were diluted with 0.4% BSA and incubated during 1 h at 37 °C. The subsequent steps of the reaction were developed as described above.

To declare a given serum sample taken during vaccination as “non-detectable” for VEGF-specific IgG1, IgG2, IgG3, or IgG4 antibodies, “specific antibody titer” must be < 1/10. Values ≥ 1/10 make samples to be classified as “detectable”. For each patient, the IgG subclass classified as “detectable” with the highest “specific antibody titer” was declared as “predominant”.

### Competitive ELISA for serum blockade of VEGF/VEGFR2 and VEGF/VEGFR1 interactions

Competitive ELISA has been previously described in details by Sánchez et al. [15]. Briefly, plates were coated overnight at 4 °C with rhVEGF. After three washes with 0.1% Tween 20 in PBS, the plates were blocked with 4% BSA for 1 h at 37 °C, followed by new washes. Serial dilutions of test sera (1/50, 1/100, 1/200, 1/400), Bevacizumab (1 μg/mL) or dilution buffer were added (100 μL/well) and incubated for 1 h at 37 °C. Then, 100 μL of 25 ng/mL of VEGFR2-Fc or 125 ng/mL of VEGFR1-Fc were added to the wells (12.5 and 62.5 ng/mL final concentration respectively) and additionally incubated for 45 min at 37 °C. After washes, wells were incubated with biotinylated anti-human VEGFR2 or VEGFR1 antibodies, the latter followed by streptavidin-peroxidase conjugate. The subsequent steps of the reaction were developed as described in previous sub-sections.

Maximum bindings of VEGFR2 or VEGFR1 were obtained from wells incubated with dilution buffer (instead of serum sample) and VEGF receptors/Fcγ chimeras (VEGFR2-Fc or VEGFR1-Fc). The inhibition caused by a given sample (sera or positive control) on VEGF/VEGFR2 or VEGF/VEGFR1 interactions was expressed as percentage, according to the following formula:

$$ \%\ \mathrm{inhibition}=100\%-\left[\left(\frac{\mathrm{absorbance}\ \mathrm{of}\ \mathrm{test}\ \mathrm{sample}}{\mathrm{absorbance}\ \mathrm{of}\ "\mathrm{Maximum}\ \mathrm{Binding}"}\right)\ *100\right]\ \left(\mathrm{C}\right) $$

Inhibition levels were expressed as a % ratio:

$$ \mathrm{inhibition}\ \mathrm{levels}=\frac{\mathrm{Post}\ \mathrm{vaccination}\ \mathrm{inhibition}\ \left(\%\right)}{ \Pr \mathrm{e}\ \mathrm{vaccination}\ \mathrm{inhibition}\ \left(\%\right)}\ \left(\mathrm{D}\right) $$

A given serum sample was considered positive for neutralizing anti-VEGF antibodies when the value resulting from this ratio was ≥2 (formula D). Patients showing at least one serum sample with neutralizing anti-VEGF antibodies during trial vaccinations or off-trial re-immunizations were considered with a positive blocking activity on the VEGF/VEGFR1 or VEGF/VEGFR2 bindings [8].

Results from competitive ELISA tests were accepted if the assay shows a variability below 10% (assay criterion). The effect of re-immunization on VEGF/VEGFR2 blockade was studied during off-trial re-immunizations. In this phase, sample “A” (before re-immunization) and sample “B” (7–10 days after re-immunization) were analyzed, where certain levels of anti-VEGF blocking antibodies could be circulating as result of monthly vaccinations. Based on assay criterion and a work published by other authors [16], a value of 10% was established as the cut off to consider an increase or not in anti-VEGF blocking activity between samples “A” and “B”.

### Measurements of platelet VEGF and sVEGFR-2 in plasma

VEGF and soluble VEGFR2 (sVEGFR-2) concentrations in serum and/or plasma samples were measured with commercially available sandwich enzyme-linked immunosorbent assay kits from R&D Systems (SVE00 and SVR200 respectively). All standard reagents and solutions, supplied by kits, were used in accordance with the manufacturer’s instructions.

VEGF released per platelet was calculated using the following formula [17]:

$$ \mathrm{Platelet}\ \mathrm{VEGF}=\frac{\left(\mathrm{Serum}\ \mathrm{VEGF}\hbox{-} \mathrm{plasma}\ \mathrm{VEGF}\right)\ x\ \left(1-\mathrm{haematocrit}\right)}{\mathrm{platelet}\ \mathrm{counts}} $$

Platelet VEGF was expressed in picograms of VEGF per million platelets. Levels of platelet VEGF and plasma sVEGFR-2 were measured at baseline (pre-vaccination), at the end of trial vaccinations (week 13) and thereafter during re-immunizations.

In the re-immunization phase, the number of available patients decreased and therefore statistical tests were not used. For each individual, platelet VEGF and sVEGFR-2 were determined in a sample taken 7–10 days after a given re-immunization. The variation of both parameters (denominated ΔVEGF or ΔsVEGFR-2) was expressed in percentage and was calculated using the following formula:

$$ \varDelta \mathrm{VEGF}\ \mathrm{or}\ \varDelta \mathrm{sVEGFR}-2=\left[\left(\frac{\mathrm{levels}\ \mathrm{after}\ \mathrm{r}\mathrm{e}\hbox{-} \mathrm{immunization}}{\mathrm{pre}\hbox{-} \mathrm{vaccination}\ \mathrm{levels}}\right)\ *100\right]-100\% $$

Based on criteria established by other authors [18], Δ ≤ -30% was considered a decrease; Δ ≥ 30% was considered an increase; -30% < Δ < 30% indicated a stability.

### Statistical analysis

All experiments included at least duplicated measurements. Data, graphs and statistic were analyzed with Graphpad Prism software version 5.0 (Graphpad Software Inc., La Jolla, CA). Differences in anti-VEGF antibodies or blocking activity were evaluated using unpaired *t*-test in pre-clinical settings. In patients, matched comparisons of platelet VEGF and plasma sVEGFR-2 from weeks 0 and 13 per treatment group, were done using paired *t* test (data that were normally distributed or after log transformation). Spearman correlation test was used to measure the correlation between one non-parametric variable with one parametric variable. Statistical significance was considered as *p* < 0.05.

## Results

### Pre-clinical research to explore the influence of dose and different adjuvants on VEGF-specific IgG antibodies and VEGF/VEGFR2 blocking activities

In order to investigate the effects on VEGF-specific antibody response of higher doses of VSSP in the VEGF vaccine formulation or the antigen combination with aluminum phosphate, we performed a pre-clinical study. This pre-clinical study was based on immunogenicity experiments done in mice, rabbits and non-human primates.

**Fig. 2:**
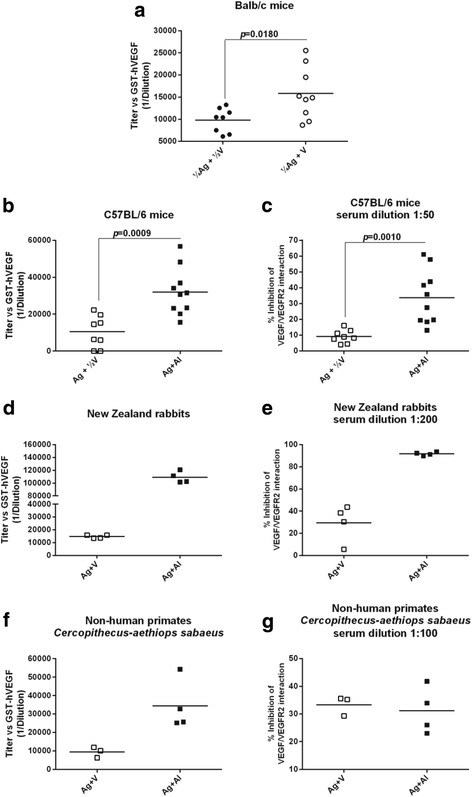
Humoral response in pre-clinical models. Specific IgG antibodies were detected by ELISA using GST-hVEGF as coating antigen (**a**, **b**, **d** and **f**). The ability of animal sera antibodies to block VEGF/VEGFR2 interaction was determined using a competitive ELISA, where a soluble VEGFR2 competes with diluted serum in plates coated with GST-hVEGF (**c**, **e** and **g**). CIGB-247 combinations using VSSP or aluminum phosphate as adjuvants were administered weekly (eight vaccinations) or bi-weekly (four vaccinations) respectively. Horizontal bars represent the mean values of antibody titer or blocking activity, which are shown for each group. *p*-Values were calculated according to unpaired *t*-test. Reference dose (A + V): 400 μg of antigen + 200 μg of VSSP; (¼Ag + ½ V): 100 μg of antigen + 100 μg of VSSP; (¼Ag + V): 100 μg of antigen + 200 μg of VSSP; (Ag + Al): 400 μg of antigen + aluminum phosphate

In mice when the amount of VSSP was doubled in the vaccine formulation (¼Ag + V), anti-VEGF antibody titers were significantly higher than those found in the group ¼Ag + ½ V (unpaired *t-test*, *p* = 0.0180) (Fig. 2a). At the same antigen dose level, the combination with aluminum phosphate elicited VEGF-specific antibody titers and VEGF/VEGFR2 blocking activities with values significantly higher than the combination with VSSP (unpaired *t-test*, *p* = 0.0009 and *p* = 0.0010 respectively) (Fig. 2b and c).

Rabbits immunized with Ag + Al showed antibody titers and blocking activities seven and three times higher than the group of rabbits vaccinated with Ag + V (Fig. 2d and e). As shown in Fig. 2 f, monkeys vaccinated with Ag + Al developed anti-VEGF antibody titers four times higher than the antibody titers seen in the group immunized with Ag + V. Only in this animal species, a similar level of blocking activity in serum was detected between both groups (Fig. 2g).

All these experimental evidences indicated that the increase of the VSSP dose or the change of adjuvant towards aluminum phosphate induce a positive effect on the humoral response specific to VEGF. For that reason, we decided to evaluate such new vaccine formulations in the framework of a clinical trial in cancer patients (CENTAURO-2).

### Patients characteristics and immunization compliance

Table 1 depicts the basic characteristics of patients included in the CENTAURO-2 clinical study. Of the fifty patients, 33 were females and 17 males (Table 1). Subjects had a variety of malignancies at original diagnosis, being the most common breast (n = 11 for a 22%), colon (n = 8 for a 16%), lung and ovary (for each one n = 7 for a 14%). At the moment of inclusion in the CENTAURO-2 trial, 96% of the patients had metastatic disease and 74% were classified as progressive disease, according the RECIST criteria. Eastern Cooperative Oncology Group performance status (ECOG) was 1 or 2 for 84% of the enrolled patients.

**Table 1:** Patients enrolled in the CENTAURO-2 phase Ib clinical trial

Characteristic	n	Percent
Age		
≥ 50	38	76%
< 50	12	24%
Sex		
Female	33	66%
Male	17	34%
Primary tumor site^a^		
Breast	11	22%
Colon	8	16%
Lung	7	14%
Ovary	7	14%
Kidney	3	6%
Uterus	3	6%
Soft tissues	3	6%
Anal canal	2	4%
Rectum	2	4%
Others	4	8%
Metastasis^b^		
Liver	13	27%
Lung	8	16%
Bone	6	12%
Lymph nodes	6	12%
Suprarenal glands	4	8%
Soft tissue	2	4%
Others	9	16%
Without metastasis	2	4%
Status^c^		
PD	37	74%
SD	13	26%
ECOG PS		
0	8	16%
1	34	68%
2	8	16%
Trial vaccinations		
Completed^d^	41	82%
No completed	9	18%

Patients were eligible for enrollment after having received available therapy and were no longer responding. ECOG PS, Eastern Cooperative Oncology Group performance status; PD, progressive disease; SD, stable disease. ^a^at the time of initial diagnosis; ^b^at the time of trial inclusion; ^c^RECIST classification at the time of trial inclusion; ^d^39 patients of the 41 were available for antibody tests and sVEGFR-2 at week thirteen; 38 patients were available for platelet VEGF

Forty-one patients completed the trial immunization scheme (82%). Of the nine non-evaluated patients, two abandoned voluntarily, other five died; one abandoned due to disease progression and one patient was excluded of the study due to the early development of brain metastasis (exclusion criteria).

### Safety

In order to investigate the safety of the different vaccine formulations, medical evaluations were done before and after each vaccination. Injection site grade I events accounted for 87.74% of all adverse events seen during the trial period (up to week 16) that could be classified as probably or definitively related to the vaccine (Additional file 1). With VSSP as adjuvant, other thirty general adverse events were also recorded, most of them grade I, exception made of one event of grade II, and two events of grade III. With aluminum as adjuvant, the majority of the adverse events were local, exception made of one case of asthenia, and all grade I. Hence, a majority of the documented adverse effects attributable to vaccination were low grade injection site events. Because of the strong bacterial contents of VSSP, the individuals immunized with VSSP vaccine formulations showed a higher amount of low grade local adverse events than when aluminum was used as adjuvant. All these events were controlled, and patients with adverse events were treated either with pharmacological or non-pharmacological therapies. All documented events happened in 29 patients (58%) of the 50 recruited individuals (Additional file 1). All deaths during trial period or re-immunization phase were attributable to the progression of their base disease.

### Antibody classes responses specific to VEGF after completion the trial vaccination scheme

To study in depth the vaccine-induced polyclonal humoral immune response, four classes of human immunoglobulins were determined by ELISAs. Of the 41 patients that finished all programmed immunizations, serum samples from 39 individuals were available for antibody tests on week 13 (one week after completion the trial vaccination scheme).

**Fig. 3:**
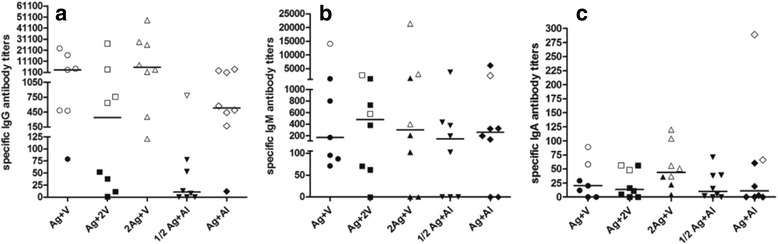
IgG (**a**), IgM (**b**) and IgA (**c**) specific antibody titers against human VEGF at week 13. Antibody titer at week 0 was subtracted from the antibody titer at week 13 (specific antibody titer). Horizontal bars represent the median values of specific antibody titer, which are shown for each group. Empty or filled symbols represent patients with positive or negative antibody samples at week 13, respectively

Figure 3a–c display specific antibody titers against VEGF for IgG, IgM, and IgA respectively, for patients of all cohorts. Each patient is represented as an empty symbol (serum sample positive for antibody at week 13) or filled symbol (serum sample negative for antibody at week 13). It can be seen that of the 39 evaluated patients, 26 individuals (66.7%) had positive samples to VEGF-specific IgG antibodies, 11 (28.2%) for IgA, and 7 (18%) for IgM. No patient had detectable levels of specific IgE (Additional file 2).

At week 13, the group 2Ag + V exhibited the highest number of samples positive for IgG or IgM or IgA antibodies specific to VEGF (Fig. 3). At this moment, there were patients with triple-positive samples (IgG/IgM/IgA). Individuals with IgG/IgA or IgG/IgM double-positive samples were detected, however, the combination IgM/IgA was not observed. All cases with single-positive samples were IgG but neither IgM nor IgA (Additional file 3).

Specific antibody titers values showed the following trend: IgG > IgM > IgA, excluding the groups Ag + 2 V and ½ Ag + Al, in which IgM antibodies specific to VEGF were higher than IgG antibodies (Fig. 3). Irrespective of the used adjuvant, VSSP or aluminum phosphate, CIGB-247 induces anti-VEGF IgG antibodies as principal class of immunoglobulins. For that reason, it was the class of immunoglobulin under study at different time points during trial vaccinations (weeks 5 or 6, 9 and 13). Vaccination increased specific IgG titers in at least one serum sample, in 43 of the 47 evaluable patients, with values as low as 1/10 and as high as 1/93981 (Additional file 4).

### Specific anti-VEGF IgG seroconversion in patients that completed the trial vaccination scheme

**Fig. 4:**
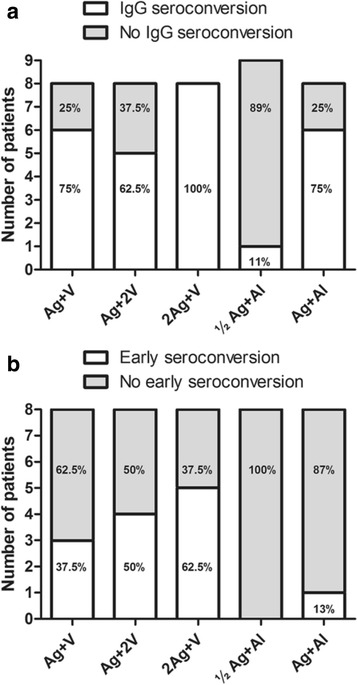
IgG seroconversion studies in patients that completed the trial vaccination scheme. **a** Seroconverted patients (individual that has shown two or more samples positive for VEGF-specific IgG antibodies), according to the different vaccination cohorts. **b** Early seroconversion: seroconverted patient with positive serum sample at week 5 (VSSP-adjuvanted cohorts) or week 6 (aluminum-adjuvanted cohorts)

In order to evaluate the seroconversion, the principal immunoglobulin class specific to VEGF was chosen for this analysis. Figures 4a depicts the results of IgG seroconversion (patient that has shown two or more samples positive for VEGF-specific antibodies) for those patients that completed the trial vaccination scheme. The reference group (Ag + V) showed six seroconverted patients out of the eight evaluable individuals (75%) (Fig. 4a). When the antigen dose was increased to 800 μg (group 2Ag + V), the proportion of seroconverted patients increased (8/8 for a 100%). The opposite effect was observed when the adjuvant VSSP was increased to 400 μg: in the Ag + 2 V group, the proportion of seroconverted patients decreased (5/8 for a 62.5%). With the same antigen dose (400 μg) and aluminum phosphate as adjuvant (group Ag + Al), the proportion of seroconverted patients was similar (6/8 for a 75%) to that of the reference group. The lowest proportion of seroconverted patients was found in the cohort vaccinated with 200 μg of antigen and aluminum phosphate as adjuvant (group ½Ag + Al) with only one patient of nine for a 11% (Fig. 4a). The percentage of seroconverted patients per cohort, showed the following order: 2Ag + V > Ag + V = Ag + Al > Ag + 2 V > ½ Ag + Al (Fig. 4a).

Early seroconversion occurs when a seroconverted patient has a positive serum sample at week 5 (VSSP-adjuvanted cohorts) or week 6 (aluminum-adjuvanted cohorts). Figure 4b shows that early seroconversion responses were preferably found in VSSP-adjuvanted cohorts. The number of early seroconverted patients for the groups 2Ag + V, Ag + 2 V and Ag + V were 5/8, 4/8, and 3/8, respectively, compared to 1/8 and 0/8 in the Ag + Al and ½Ag + Al cohorts.

### VEGF/VEGFR2 and VEGF/VEGFR1 blocking activities in patients that completed the trial vaccination scheme

VEGF and its receptors VEGFR1 and VEGFR2 play major roles in tumor angiogenesis [19]. In order to assess the ability of serum antibodies from a vaccinated patient to block the binding of VEGF to its receptors, a competitive ELISA was performed.

**Fig. 5:**
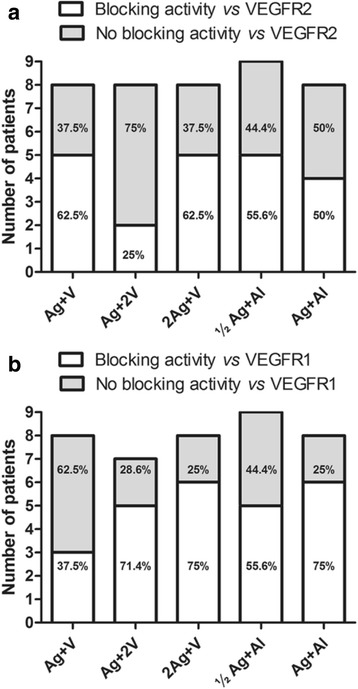
VEGF/VEGFR2 and VEGF/VEGFR1 blocking activities in patients that completed the trial vaccination scheme. **a** VEGF/VEGFR2 blocking activity according to the different vaccination cohorts. **b** VEGF/VEGFR1 blocking activity according to the different vaccination cohorts. Patients that has shown at least one serum sample with neutralizing anti-VEGF antibodies were considered with a positive blocking activity on the VEGF/VEGFR1 or VEGF/VEGFR2 bindings

Figure 5a shows the number of patients with positive blocking activity on the VEGF/VEGFR2 binding. Individuals showing at least one serum sample with neutralizing anti-VEGF antibodies were considered positive for blocking activity. In the reference group, five out of eight patients (62.5%) had a positive blocking activity on the VEGF/VEGFR2 binding, similar to the group 2Ag + V. A slightly lower number of positive patients were observed for the aluminum-adjuvanted cohorts: ½Ag + Al (5/9 for a 55.6%) and Ag + Al (4/8 for a 50%). The lowest number of positive patients was found in the Ag + 2 V group (2/8 for a 25%).

Figure 5b depicts a similar analysis for the VEGF/VEGFR1 blocking activity test. The reference group (Ag + V) exhibited the lowest number of positive patients (3/8 for a 37.5%). When the amount of antigen or VSSP were doubled as compared to the reference group, the proportion of positive patients increased in both groups, (6/8 for a 75%) and (5/7 for a 71.4%), respectively. In the aluminum- adjuvanted cohorts, the rates were 6 out of 8 patients for the Ag + Al group (75%), and 5 out of 9 patients for the ½Ag + Al group (56%).

All results shown above demonstrated that using of aluminum as adjuvant, a higher humoral response was obtained at the antigen dose level of 400 μg. Of all antigen doses and adjuvant combinations, the most immunogenic dose was 800 μg of antigen in combination with 200 μg of VSSP.

### IgG seroconversion and VEGF/VEGFR2 blocking activity in patients that received off-trial re-immunizations

One of the features of this study is the relatively long period over which the patients were immunologically evaluated (up to week 60), receiving up to eleven re-immunizations (Additional file 5). Re-immunizations were administered every four weeks. Patients belonging to groups Ag + V and 2Ag + V conserved their original vaccine formulation in this phase of the study. Patients from the Ag + 2 V, ½Ag + Al and Ag + Al cohorts kept their original vaccine formulation for re-immunization, until the approval of the final trial report by CECMED. At this point, taking into consideration the results of safety and humoral response, these three cohorts switched to 800 μg of antigen + 200 μg of VSSP, always under medical supervision. For each patient, the time point of dose change is shown in Additional file 5. Because of their distinct recruitment moment, the exact time point of the switch was different between individuals, even in a same cohort.

Of the thirty-two patients that were eligible for off-trial re-immunizations, twenty-two individuals had at least two serum samples after week sixteen (Additional file 5). In these patients, studies involving seroconversion and VEGF/VEGFR2 blocking activity were done. VEGF/VEGFR1 blockade was not studied.

Of these 22 patients, 16 seroconverted individuals (72.7%) during trial vaccinations conserved their status during re-immunization phase. Three patients (13.6%) with no evidence of seroconversion turned to seroconverted status after off-trial re-immunizations. Two individuals (9.1%) remained negative in both phases and one patient (4.5%) lost his seroconverted status. In the case of VEGF/VEGFR2 blocking activity, eleven individuals (50%) with positive blocking activity during trial vaccinations conserved their status during re-immunization phase. Five patients (22.7%) with no documented blocking activity turned to positive status after off-trial re-immunizations. Three individuals (13.6%) remained negative in both phases and another three patients lost their positive blocking activity during re-immunization phase.

### Evolution of VEGF-specific IgG antibodies and VEGF/VEGFR2 blockade in twelve patients submitted to long-term re-immunizations

**Fig. 6:**
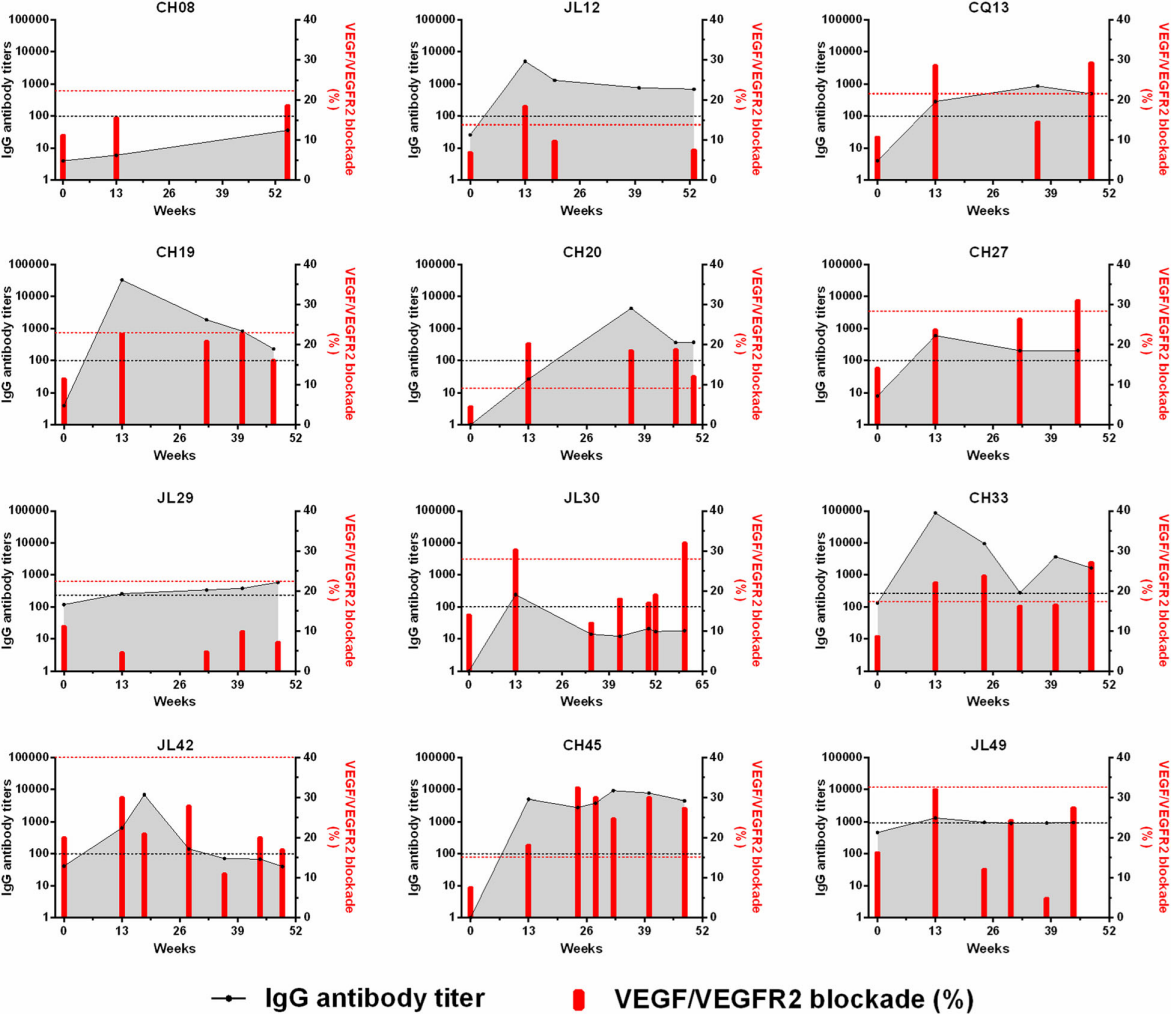
Evolution of VEGF-specific IgG antibodies and VEGF/VEGFR2 blockade in patients submitted to long-term re-immunizations. Samples, taken at different time points, came from twelve patients (depicted by their trial code name) with an overall survival longer than 45 weeks (approximately 1 year). Antibody titers and VEGF/VEGFR2 blockade percentages are shown as *black dots* and *red bars*, respectively. Week 0 is pre-vaccination and week 13 is one week after finishing the trial vaccinations. Cut-off values that define the positivity for IgG antibodies (*black discontinued line*) and VEGF/VEGFR2 blockade (*red discontinued line*) are shown for each patient

Twelve of the thirteen patients submitted to long-term re-immunizations with an overall survival longer than 45 weeks (approximately 1 year) were studied. In these patients, evolution of VEGF-specific IgG antibodies and VEGF/VEGFR2 blockade were studied in more detail, including their pre-vaccination values (week 0) and values at week 13 (one week after the last immunization of the trial vaccinations). All these patients were individually followed in Fig. 6.

For most patients, serum samples obtained during re-immunization phase had VEGF-specific IgG antibodies titers higher than pre-vaccination, regardless of the fact that samples could be considered positive or negative (Fig. 6). Only in patient JL42, IgG antibodies gradually decreased to pre-vaccination levels at week 50. Taking as reference the end of trial vaccinations (week 13), anti-VEGF IgG antibody titers presented an increase or a decrease during the re-immunization phase. Regarding positivity of anti-VEGF IgG antibody titers, eight patients (JL12, CQ13, CH19, CH27, JL29, CH33, CH45 and JL49) had positive serum sample at week 13 and then maintained their positivity in samples taken during re-immunization period. One subject (CH20), with negative serum sample at week 13, showed for the first time, positivity for three consecutive serum samples. Another patient (JL30), with positive serum sample at week 13, lost this status in five consecutive serum samples. Patient JL42 had a positive serum sample at week 13, conserved this status in samples of weeks 18 and 28, and then lost his positivity in samples of weeks 36, 44 and 49. Finally, patient CH08 did not exhibit positivity in any of the times when the serum sampling was made.

The VEGF/VEGFR2 blockade, expressed in percentage, is characterized by a very fluctuating behavior during the course of sustained immunization (Fig. 6). Concerning positivity for VEGF/VEGFR2 blockade, Fig. 6 shows that one patient (CH27) with negative serum sample at week 13, increased the percentage values at week 32, being positive at week 45. Two individuals (CH45 and CH20) had a positive serum sample at week 13, and all serum samples taken during off-trial re-immunization maintained their positivity. Three patients (CQ13, JL30 and CH33) were positive at week 13, and during the re-immunization phase these patients restored their positive status after having lost it. In two other patients (JL12 and CH19), samples taken during the re-immunization phase did not show the positive status that have been present in week 13. Finally, serum samples for four patients (CH08, JL29, JL42 y JL49) never reached values above cut-off and remained negative.

### Re-immunizations effects on VEGF-specific antibodies and VEGF/VEGFR2 blockade in twelve patients submitted to long-term re-immunizations

All these aforementioned findings could be influenced by the moment in which the sample was taken. The samples available during off-trial re-immunizations were mostly acquired at the moment of a new vaccination, i.e., approximately four weeks after a previous re-immunization. However, it is well known that the earliest time at which serum antibody peaks following vaccination is at about 7–14 days. To better study the effect of re-immunization on anti-VEGF antibody levels and VEGF/VEGFR2 blockade, blood samples from 12 patients submitted to long-term vaccinations were taken just before the re-immunization (sample A) and then, these patients were summoned 7 to 10 days after this re-immunization for additional blood sampling (sample B). Table 2 shows the study results for IgG, IgM and IgA antibodies specific to VEGF, as well as VEGF/VEGFR2 blockade in these serum samples. Re-immunization was considered effective if: i) sample “B” conserves or gains positivity or ii) value in sample “B” is higher than value obtained in sample “A” (*antibody titer: sample “B” - sample “A”* ***≥*** *1/100 or VEGF/VEGFR2 blockade: sample “B” - sample “A”* ***≥*** *10%*).

**Table 2:** Effect of the re-immunization on VEGF-specific IgG, IgA and IgM antibodies or VEGF/VEGFR2 blockade

Patient code	IgG antibody titer			IgA antibody titer			IgM antibody titer			VEGF/VEGFR2 blockade (%)		
	Pre-vaccination	Re-immunization phase		Pre-vaccination	Re-immunization phase		Pre-vaccination	Re-immunization phase		Pre-vaccination	Re-immunization phase	
	Week 0	Sample A	Sample B	Week 0	Sample A	Sample B	Week 0	Sample A	Sample B	Week 0	Sample A	Sample B
CH08	4	36	31	49	70	71	1183	1035	1101	11.2	18.7	11.9
JL12	26	700^*^	770^*^	49	93	56	298	528	604^*^	6.92	7.5	15.2^*^
CQ13	4	505^*^	582^*^	335	286	305	1655	2090	2198^†^	10.8	29.4^*^	24.5^*^
CH19	4	232^*^	244^*^	49	59	68	1180	1462	1978^†^	11.5	16.1	31.5^*,#^
CH20	1	374^*^	339^*^	110	94	62	963	629	675	4.6	12.1^*^	16.7^*^
CH27	8	207^*^	236^*^	268	472	319	1947	3422	3732^†^	14.2	31.1^*^	29.9^*^
JL29	118	589^*^	795^*,†^	87	124	144	707	438	557^†^	11.2	7.2	20.8^#^
JL30	1	17	17	69	72	75	886	663	688	14	19	18
CH33	133	1655^*^	1697^*^	111	676^*^	530^*^	2591	341140^*^	3859811^*,†^	8.7	27.2^*^	28.9^*^
JL42	41	40	56	70	73	96	1788	1374	2426^†^	20	17	27^#^
CH45	0	4474^*^	3825^*^	48	80	84	543	4108^*^	4995^*,†^	7.6	27.4^*^	23^*^
JL49	463	947^*^	1110^*,†^	70	155^*^	125	509	1114^*^	1027^*^	16.3	27.5	34.1^*^

Sample “A”: is the value for the sample taken just before the re-immunization; Sample “B”: is the value for the sample acquired 7–10 days after the taking of the sample “A”. [*]: sample meets the positivity criteria for specific-IgG (using formulas A and B), IgA or IgM antibodies (using formula A) or VEGF/VEGFR2 blockade (using formula D). (**†**): sample meets the criterion of increase for antibody titer: sample “B” - sample “A” ≥ 1/100. (**#**): sample meets the criterion of increase for VEGF/VEGFR2 blockade: sample “B” - sample “A” ≥ 10%. Patients: CH08 (sample A taken at week 55; sample B taken at week 56); JL12 (sample A taken at week 53; sample B taken at week 54); CQ13 (sample A taken at week 48; sample B taken at week 49); CH19 (sample A taken at week 47; sample B taken at week 48); CH20 (sample A taken at week 50; sample B taken at week 51); CH27 (sample A taken at week 45; sample B taken at week 46); JL29 (sample A taken at week 48; sample B taken at week 49); JL30 (sample A taken at week 52; sample B taken at week 53); CH33 (sample A taken at week 48; sample B taken at week 49); JL42 (sample A taken at week 49; sample B taken at week 50); CH45 (sample A taken at week 48; sample B taken at week 49); JL49 (sample A taken at week 44; sample B taken at week 45)

Regarding positivity of anti-VEGF IgG antibody titers, nine of the twelve patients (JL12, CQ13, CH19, CH20, CH27, JL29, CH33, CH45 and JL49) with the re-immunization conserved their positivity for the test. Samples “A” and “B” were positive in all cases (Table 2). For IgM, the re-immunization was effective in four patients (JL12, CH33, CH45 and JL49). In the case of patient JL12, had a negative sample “A”, after the re-immunization, he gained positivity in sample “B”. For IgA, only patient CH33 conserved positivity (both samples “A” and “B” were positive). Concerning VEGF/VEGFR2 blockade, the re-immunization was effective in eight of the twelve patients. Of these eight patients, five individuals (CQ13, CH20, CH27, CH33 and CH45) had positive results both in sample acquired at re-immunization time (sample “A”), and 7–10 days afterwards (sample “B”). The remaining three individuals (JL12, CH19 and JL49) had sample “A” classified as negative, and they changed to positive in the sample taken later (sample “B”) (Table 2).

The effect of re-immunization on antibody levels was studied in the same cohort of patients underwent long-term vaccinations. In general, the re-immunization had a limited effect on specific-IgG or IgA antibody levels. For IgG, only two individuals increased their IgG antibody titers, from 1/589 (sample “A”) to 1/795 (sample “B”) in patient JL29 and from 1/947 to 1/1110 in patient JL49. For IgA, no increments were detected. A different scenario was seen for IgM, with seven patients (CQ13, CH19, CH27, JL29, CH33, JL42 and CH45) showing IgM antibody titers in sample “B” higher than the values obtained in sample “A” (Table 2). In the case of VEGF/VEGFR2 blockade, in three patients (CH19, JL29 and JL42), inhibition percentages were found to be higher in sample “B” than the values obtained in sample“A”.

### IgG subclasses

**Fig. 7:**
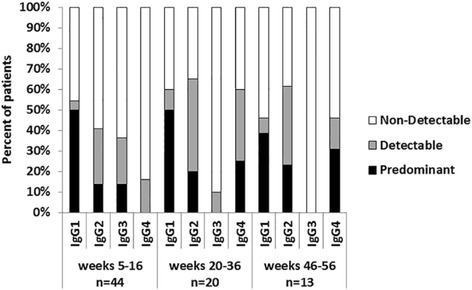
Percentages of patients with different VEGF-specific IgG subclasses in the weeks 5–16, 20–36 and 46–56. In each of these stages, and for the available patients, the study was made in the sample with the highest specific IgG antibody titer. “n” represents the number of evaluated patients. Terms “non-detectable”, “detectable” and “predominant” are detailed in Methods

As was demonstrated previously, anti-VEGF IgG antibodies were the principal class of immunoglobulins. In order to study the contribution of each one of the four VEGF-specific IgG subclasses, indirect ELISA was performed using human VEGF as coating antigen. Figure 7 shows IgG subclasses analysis without regarding antigen doses or vaccination schedules. The study was made for three different vaccination periods or stages: weeks 5–16 (trial period), weeks 20–36 (early off-trial re-immunizations) and weeks 46–56 (long-term re-immunizations). In each of these stages, and for the available patients, serum samples with the highest anti-VEGF IgG antibody titers were chosen for these measurements.

IgG1, IgG2 and IgG4 subclasses specific to VEGF were found in all periods. Only IgG3 was not detected during long-term re-immunizations (Fig. 7). The predominant subclass in all periods was IgG1, accounting for 50% of patients in the first two stages, and 38.5% in the third and last. Both, IgG2 and IgG3, were the second most important immunoglobulins during trial period with 13.6% of the patients. IgG2 was conserved over time as “detectable” or as “predominant” subclass. IgG3 had a tendency to disappear. Finally, IgG4 increased over time as “predominant” subclass from 0% in trial period to 30.8% of the patients during long-term re-immunizations, being the second most relevant IgG subclass during this latter period.

### Platelet VEGF and plasma levels of sVEGFR-2

VEGF and sVEGFR-2 have been extensively studied in several anti-cancer or anti-angiogenesis treatment strategies [20–23]. In order to evaluate the dynamic changes on platelet VEGF and sVEGFR-2 during vaccination with CIGB-247, we measured the baseline levels (week 0 or pre-vaccination), at the end of the trial vaccinations (week 13) and approximately 1 year after initial immunization.

Table 3 shows that a statistically significant reduction on platelet VEGF values with vaccination only occurred in the groups 2Ag + V (*p = 0.0244*) and Ag + Al (*p = 0.0086*). No change was observed on plasma levels of sVEGFR-2 for any of the immunization groups.

**Table 3:** Comparison of platelet VEGF and plasma sVEGFR-2 per treatment groups in the CENTAURO-2 trial

Groups	pg of VEGF/10^6^platelets Mean (range) [n]		pg/mL of sVEGFR-2 Mean (range) [n]	
	Week 0	Week 13	Week 0	Week 13
Ag + V	1.21 (0.30–2.20) [7]	0.61 (0.10–1.43) [7] *ns*	9736 (6939–11724) [7]	9792 (7427–11162) [7] *ns*
Ag + 2 V	0.93 (0.55–1.46) [7]	1.01 (0.08–3.29) [7] *ns*	10455 (8116–12653) [8]	10423 (7262–12882) [8] *ns*
2Ag + V	2.07 (0.11–8.19) [8]	0.57 (0.22–2.05) [8] p = 0.0244*	9736 (7078–12637) [8]	9948 (9125–11098) [8] *ns*
½Ag + Al	1.90 (0.90–3.64) [8]	1.98 (0.63–3.54) [8] *ns*	10228 (7555–12735) [8]	9567 (7820–13372) [8] *ns*
Ag + Al	1.58 (0.72–3.19) [8]	1.06 (0.15–2.91) [8] p = 0.0086**	10131 (8491–12223) [8]	10296 (8611–13294) [8] *ns*

The table summarizes the data per vaccine dose cohorts and the results of paired *t*-test (*ns*: non-significant; *p < 0.05; **p < 0.01). *(n)* number of patients

The relationship between the variation of platelet VEGF (ΔVEGF), and specific IgG antibodies titers (week 13 – week 0), was studied in 38 patients disregarding the vaccine dose and scheme. An inverse and statistically significant correlation was observed (Spearman correlation coefficient, *r* = -0.5888, *p < 0.0001*) indicating that patients with higher specific IgG antibody titers decreased their platelet VEGF levels. However, there were not statistically significant correlations between ΔVEGF and IgA antibody titers (*r* = -0.1979, *p* = 0.2337) or ΔVEGF and IgM antibody titers (*r* = -0.2145, *p =* 0.1960).

**Fig. 8:**
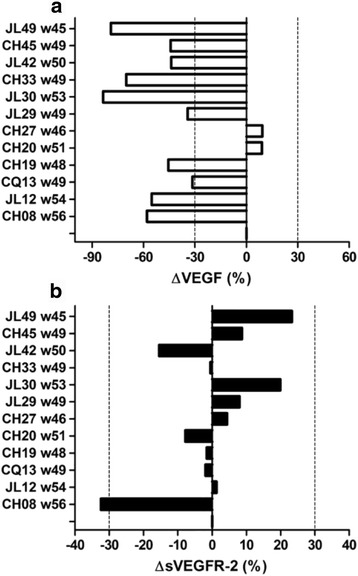
Changes in platelet VEGF and plasma sVEGFR-2. Platelet VEGF (**a**) and plasma sVEGFR-2 (**b**) were expressed in percentages relative to baseline levels (week 0 or pre-vaccination). Discontinued lines represent the cut-off values that indicate: ≥30% increase; ≤-30% decrease; between 30% and -30% stability. (w): is the week when the sample “B” was taken (7–10 days after given re-immunization)

Twelve of the thirteen patients submitted to long-term re-immunizations with an overall survival longer than 45 weeks (approximately 1 year) provided blood samples. (Figure 6). Serum and plasma samples were collected, 7-10 days after a given re-immunization time point (time point for the taking of sample “B”, see Fig. 8 for specific week). These patients were checked for platelet VEGF (Fig. 8a) and sVEGFR-2 (Fig. 8b). Most patients (10/12 for an 83%) had platelet VEGF levels lower than baseline (ΔVEGF ≤ -30%, decrease of platelet VEGF). The remaining two patients (2/12 for a 17%), showed stability in platelet VEGF (-30% < ΔVEGF >30%). For sVEGFR-2, most patients (11/12 for a 92%) had no change and only one individual (1/12 for an 8%) had levels of sVEGFR-2 lower than baseline.

## Discussion

So far CENTAURO and CENTAURO-2 clinical trials show the only available results worldwide for a VEGF active immunization procedure in humans. The novelty of our VEGF-based vaccine makes difficult to find comparable settings in the clinical practice. A similar therapeutic vaccine that uses a VEGF peptide in combination with the adjuvant RFASE is being investigated in a phase I clinical trial (NCT02237638), but this study is still recruiting patients and no results are available yet. Thus, we focus our comparisons in the results found in this trial (CENTAURO-2) and the previous one (CENTAURO) [8]. We also discuss some pre-clinical experiments to bridge the gap between the two clinical trials. Additionally, we compare our results with cancer vaccines directed to other self-antigens.

The excellent safety results of the CIGB-247 vaccine candidate (using VSSP as adjuvant), together with its ability to induce specific anti-VEGF antibodies able to block the interaction between VEGF and VEGFR2, were among the main hallmarks of the CENTAURO trial [8]. The CENTAURO-2 phase Ib clinical trial was designed to test new vaccine compositions, incorporating higher antigen doses in combination with the adjuvants VSSP or aluminum phosphate.

Evidences gathered in a majority of the enrolled patients showed that vaccination with different CIGB-247 formulations exhibited a very positive safety profile. This is in line with the fact that it is generally recognized that many cancer vaccines have few adverse reactions [24].

Some clinical studies on cancer vaccines have revealed a relationship between the high levels of elicited antibodies and an improved survival; and even have been able to elucidate the specific immunoglobulin class associated with overall survival [25–27]. Although these types of correlations are not applicable to phase I clinical trials, there is no doubt about the importance of studying in depth the humoral response in early evaluations of cancer vaccines in humans [24]. A special emphasis was done in this paper in the characterization of the vaccine-induced humoral response in terms of quantity, quality, kinetic, dynamic and composition.

An important goal of this study (CENTAURO-2) was to determine if CIGB-247 antigen in different settings of adjuvants and higher doses of antigen or adjuvant is capable to elicit a specific humoral response against human VEGF higher than the response seen in CENTAURO clinical trial [8].

The reference Ag + V group and the Ag + Al cohort had similar seroconversion rates, but unexpectedly, the former showed faster (earlier) seroconversion and also developed higher titers of specific IgG antibodies. An explanation to this difference has to consider first the use of different adjuvants. While aluminum has been employed by others in cancer patient vaccination [28, 29], the bacterially-derived VSSP has previously shown to be a very strong stimulator of specific humoral responses in cancer patients [30, 31], and its use in our case may favor the speed and intensity of the response against VEGF. The second aspect that could explain these differences is the total amount of antigen administered, which is much lower in the aluminum cohort due to the bi-weekly vaccination scheme. If this is true, the results differ substantially from those of our pre-clinical studies done in mice, rabbits, and non-human primates, where using bi-weekly immunization schedules with the VEGF antigen formulated in aluminum produced higher specific IgG antibody titers or VEGF/VEGFR2 blocking activities, with respect to control groups immunized weekly with the same antigen dose per injection, and VSSP as adjuvant. Irrespectively of the fact that we do not have at present a mechanistic explanation for these differences in the behavior of pre-clinical models and human cancer patients, our findings illustrate how carefully extrapolations between them should be done.

Another dissimilar result between pre-clinical models and cancer patients was found when the amount of VSSP was doubled in the vaccine formulation. Although in mice a doubled dose of VSSP led to an increase in anti-VEGF antibody titers, in cancer patients, a lower amount of IgG seroconverted individuals was observed in the Ag + 2 V cohort as compared with the Ag + V group. This result could be due to the high dose of VSSP, which has a negative effect on humoral response only in humans. The amount of powerful bacterial antigens present in this dose of VSSP could be competing against VEGF for a humoral response, a phenomenon known as antigenic competition. In fact, VSSP is known to induce high levels of anti-VSSP antibodies in cancer patients [30, 31]. Although the actual mechanisms of antigenic competition are still not fully understood, it is well established that the degree of competition increases as the dose of the competing antigen is increased [32, 33].

The results of CENTAURO-2 study show that vaccinating with VEGF, combined either with VSSP or aluminum phosphate, leads to the production of specific anti-VEGF IgG, IgM, and IgA antibodies. These three types of immunoglobulins elicited in patients by using other cancer vaccines based on defined specific tumor antigens have demonstrated complement dependent cytotoxicity (CDC) and antibody dependent cellular cytotoxicity (ADCC) activities [27, 34, 35]. However, these effector mechanisms of antibodies are typical when their respective antigens are membrane surface proteins. In our case, VEGF is a soluble protein, and together with IgG, we can now add a possible role of IgM and IgA to the potential of the antibody response elicited by CIGB-247 in blocking the interactions of VEGF and VEGF receptors. Additional to ligand depletion, FcγR–mediated enhancement of antigen presentation is another mechanism contributing to tumor immunity [36]. It has been reported that immune complexes, formed as a consequence of IgG antibody binding to its antigen, can enhance in dendritic cells antigen uptake and upregulate antigen presentation, both to MHC class II-restricted CD4^+^ T cells and to CD8+ T cells [37, 38]. It is being increasingly recognized that IgG immunoglobulin are potent integrators of innate and cellular immunity or a link between humoral and cellular immunity, resulting in increased immune responses [39]. Similar mechanisms have been described for IgM and IgA antibodies via Fcα/μR (Fc receptor for IgM and IgA immunoglobulins) or FcαRI [40, 41].

For a vaccine candidate targeting a growth factor relevant for tumor growth is imperative to test not only the specific antibody response elicited against VEGF but also the ability of such antibodies to block the binding of VEGF to VEGFR2 or VEGFR1. Because of the aforementioned, VEGFR2 and VEGFR1 blocking activities documented in serum samples from CENTAURO-2 patients deserve special attention. In particular, the blockade of VEGF/VEGFR1 interaction had not been studied before with our vaccine candidate, and these results add another possible mechanism to others involved in the final anti-tumor potential of CIGB-247. The humoral response induced by CIGB-247 vaccine, could be able to impair VEGF/VEGFRs axis that mediates important processes for tumor development including tumoral angiogenesis and tumor-induced immunosuppression [19, 42].

The second part of this discussion will be devoted to the analysis of the humoral response results in patients from the CENTAURO-2 trial that received off-trial monthly re-immunizations. In the field of cancer therapeutic vaccines, chronic vaccination is regarded as essential, especially when the vaccines involve self-antigens [43]. Immunizations may boost pre-vaccination anti-VEGF antibodies detected in some patients, improving it in terms of quantity and quality. This particular effect could be more relevant for patients who already naturally have anti-VEGF blocking antibodies.

Regarding the assessment of boost vaccination effect in patients with longer survival and baseline anti-VEGF blocking antibodies, an ideal situation could be the one where the antibody assessment during off-trial monthly re-immunizations could be controlled by the inclusion a non-vaccinated patients in order to determine whether this is specific to the vaccination administered or whether this post-vaccination blocking activity is found naturally in these patients. It is not feasible to obtain control samples from non-treated patients in this clinical trial because of three major reasons: (a) patients of CENTAURO-2 trial had a variety of malignancies, for that reason the non-vaccinated patients should be representative of this heterogeneity; (b) most of the patients of CENTAURO-2 trial had metastatic disease and were classified as progressive disease according the RECIST criteria. The non-vaccinated patients should be representative of this advanced illness, where a survival greater than 1 year is a big challenge; (c) patients were eligible for enrollment in this clinical trial after having received available therapy and were no longer responding. Thus, it is not ethically correct that a group of patients without treatment options were not eligible for the vaccine candidate, even more, when in our hands we have data about patients with advanced illness and immunized with the vaccine candidate able to show objective clinical benefits [8, 44]. Additionally, it is well studied that patients in frank progression have a strong tumor-induced immunosuppression [45, 46]. This immunosuppression has a negative impact on the cellular and humoral responses. In this context, the probability of increasing a basal blocking activity over time is quite low. Therefore, the increase of the basal blocking activity over time observed in these longer survival patients can only be explained by the monthly re-immunizations.

Despite the limitations imposed from the switch of patients from their original vaccine formulation to that of 800 μg of antigen + 200 μg of VSSP during off-trial re-immunizations, with respect the scope of the conclusions to be drawn, the off-trial vaccination phase and patient follow up allowed us to document that re-immunization was safe. Additionally, re-immunization was relevant for a number of patients, in terms of helping to maintain positive specific IgG antibody titers and/or VEGF/VEGFR2 blocking ability, or eventually achieving a seroconversion or VEGF/VEGFR2 blocking status, not documented during the trial period, or lost thereafter. These results are in line with our findings in the preceding CENTAURO clinical study [8], and other follow up studies about the vaccine candidate in combination with VSSP [44, 47].

Continued vaccination was also important to produce a gradual shift in the anti-VEGF IgG response from IgG1 to IgG4. IgG1/IgG4 subclass switching has been previously reported using a CEA-based vaccine in colorectal cancer patients [48]. IgG4 antibodies are prominent only after prolonged immunization with protein antigens, and have been associated with the affinity maturation process [49]. They are considered the highest affinity antibodies that could lead to more efficient antigen neutralization [50]. The potential generation of VEGF-specific antibodies of much greater affinity relative to those obtained during the trial vaccination scheme is highly relevant because of the high affinity of the interaction between VEGF and VEGFR2 (Kd = 37x10^−12^ M) [51]. The presence of these high-affinity antibodies requires further investigation by surface plasmon resonance.

We found that re-immunizations during off-trial vaccinations increased specific anti-VEGF IgM antibody titer. While IgM is commonly associated with a primary immune response after initial exposure to an antigen, Seifert et al*.* have also found IgM memory B cells that are generated in T cell-dependent immune responses, with similar features of class-switched memory B cells: enhanced antigen response, proliferation, increased metabolic turnover, a propensity to plasmablast differentiation and a higher and faster reactivity [52]. This experimental evidence could explain the presence of specific IgM antibodies during long-term vaccination with CIGB-247.

VEGF is a soluble factor and platelets are considered one of the most important physiological transporters of VEGF [53]. Blood platelets have an active role on tumor angiogenesis and metastasis formation [54]. All these elements suggest that VEGF content within platelets may be a meaningful interesting potential biomarker for studying the effect of VEGF-based immunotherapies.

In the preceding CENTAURO clinical trial, we have documented that only the cohort that received the highest antigen dose (400 μg antigen + 200 μg VSSP) showed statistically significant reduced levels of platelet VEGF, with respect to pre-vaccination values [8]. However, in CENTAURO-2 study this drop in platelet VEGF was not observed in the group with the same dose and schedule (reference group). These apparently contradictory results in the two trials could be related to differences in the types of tumors, localization and stages. Another element to always consider is the relatively low number of individuals per group, which limits the scope of the results and the extension of them from one study to another. Our findings strongly support that elicited antibodies specific to VEGF in different adjuvants settings do not only block the interaction with its receptors but also reduce in vivo platelet VEGF bioavailability. Such results shed some light on the mechanism of CIGB-247 anti-tumoral effects by adding antigen sequestration capabilities to the already described specific neutralization of the ligand interaction with the receptor.

In this work, we found a statistically significant correlation between IgG response and the variation of platelet VEGF when pooling all trial patients. Not many cancer therapeutic vaccines have been developed using soluble growth factors as target antigens, but a result that is in line with our findings is that reported by Rodríguez et al. in a phase III study of CIMAvax-EGF, a therapeutic vaccine for the treatment of patients with non-small cell lung cancer, where EGF is the antigen. These authors also found a significant inverse correlation between the anti-EGF antibody titers and serum EGF concentration [55].

Modulation of the soluble version of membrane VEGFR2 (sVEGFR-2) has been extensively studied after treatment with tyrosine kinase inhibitors (TKIs) or with single-agent Bevacizumab, a humanized monoclonal antibody specific to human VEGF. Patients treated with TKIs have decreased levels of sVEGFR-2 [20, 21, 56, 57]; however Bevacizumab induces the opposite effect (i.e., an increase in sVEGFR-2 levels) [22, 23, 58]. A different modulation profile regarding sVEGFR-2 has been ascribed to receptor blockade with TKIs or direct ligand depletion with Bevacizumab.

The sVEGFR-2 levels were not studied in the CENTAURO trial. In the CENTAURO-2 clinical trial the majority of the patients, both during the trial, and off-trial, did not show any significant changes with respect to pre-vaccination levels. Bevacizumab and antibodies elicited by CIGB-247 possibly share VEGF neutralizing antibodies as part of their potential anti-tumor mechanisms; however they did not produce the same effect. In fact, specific immunoglobulin concentrations in blood after therapy onset are dissimilar for these two different therapeutic strategies (active immunotherapy *versus* passive immunotherapy).

In order to explain this difference, we will be based on the theory presented by Loupakis et al., which indicates that the increase of sVEGFR-2 levels on Bevacizumab-containing therapies may be related to the switch on of activated endothelial cells or progenitors of the tumor’s microenvironment [59]. This possible tumor and host-driven resistance mechanism could be caused by induced stress due to the high dose of Bevacizumab administered during infusion. However, in the context of vaccination with CIGB-247, levels of vaccine-induced antibodies are not so extremely high as to cause this phenomenon. This type of strategy could be seen as a sort of metronomic therapy with low levels of elicited antibodies as compare with intravenous administration of Bevacizumab. At these lower specific antibody levels, both toxicity for normal tissues and the induction of sVEGFR-2 increase are most probably not favored.

Taking into account the results of all ELISA tests described here, with aluminum as adjuvant, a further increase in antigen dose over 400 μg is foreseen in order to achieve a higher specific humoral response. The best results of humoral response seen at the dose level of 800 μg of antigen indicate the potential use of this dose in combination with either VSSP or aluminum phosphate as adjuvants.

## Conclusions

The present study shows that vaccination with CIGB-247 at different antigen doses and in combination with different adjuvants, is safe, and induces predominantly IgG, but also IgM, and IgA antibodies specific to human VEGF. Elicited antibodies also block the interaction between VEGF and its receptors VEGFR1 and VEGFR2. Vaccination with CIGB-247 is associated with a depletion of platelet VEGF. All these properties are preserved with monthly immunizations up to 1 year. Particularly, as immunizations number increases, anti-VEGF IgG response shifts gradually from IgG1 to IgG4, being the former the predominant subclass. Both strategies using either VSSP or aluminum phosphate as adjuvants combined with the highest dose of antigen (800 μg) deserve further evaluations in phase II clinical trials.

## Additional files

Additional file 1: Reported adverse events which were probably or definitively related to the vaccine during trial period. (PDF 113 kb)

Additional file 2: IgE antibody titers specific to human VEGF. (XLSX 13 kb)

Additional file 3: Detection of specific-IgG, IgM or IgA immunoglobulin classes at the end of trial vaccinations (week 13). (PDF 100 kb)

Additional file 4: Anti-VEGF IgG antibody titers prior and after vaccination during trial vaccinations. (XLS 41 kb)

Additional file 5: Off-trial re-immunizations. (PDF 97 kb)
