# Supplementary material for: Characteristics of the specific humoral response in patients with advanced solid tumors after active immunotherapy with a VEGF vaccine, at different antigen doses and using two distinct adjuvants
Source: BMC Immunol. 2017 Jul 26;18:39. doi: 10.1186/s12865-017-0222-z (PMC5530503; doi:10.1186/s12865-017-0222-z)
Supplement: Supplementary file 1 — Reported adverse events which were probably or definitively related to the vaccine during trial period. (PDF 113 kb) [file 12865_2017_222_MOESM1_ESM.pdf]

Additional file 1. Reported adverse events which were probably or definitively related to the vaccine during trial period.

Cohort	Ag+V		Ag+2V			2Ag+V	½Ag+Al	Ag+Al	Totals
Event/Grading	I	III	I	II	III	I	I	I	
IS erythema	19	0	42	0	0	31	10	20	122
IS pain	9	0	17	0	0	24	6	11	67
IS lump	1	0	7	0	0	4	5	5	22
IS warmth	1	0	0	0	0	2	0	1	4
IS edema	1	0	1	0	0	0	1	0	3
IS pruritus	1	0	1	0	0	0	0	0	2
IS weal	0	0	0	0	0	1	0	0	1
IS paresthesia	1	0	0	0	0	0	0	0	1
Asthenia	0	1	0	0	0	0	1	0	2
Fever	1	0	4	0	0	2	0	0	7
Malaise	5	0	1	0	0	0	0	0	6
Pseudo flu	0	0	2	0	0	2	0	0	4
Chills	0	0	0	0	0	1	0	0	1
Allergic reaction	0	0	0	1	0	0	0	0	1
Headache	0	0	0	0	0	1	0	0	1
Hypertension	0	0	0	0	1	2	0	0	3
Tachycardia	0	0	1	0	0	0	0	0	1
Arthralgia	3	0	0	0	0	0	0	0	3
Bone pain	0	0	0	0	0	1	0	0	1
Myalgia	0	0	0	0	0	1	0	0	1
Totals	42[6]	1[1]	76[7]	1[1]	1[1]	72[7]	23[4]	37[5]	253[29]

IS, injection site; number of individual patients with the adverse event in square brackets.
