# Supplementary material for: Characteristics of the specific humoral response in patients with advanced solid tumors after active immunotherapy with a VEGF vaccine, at different antigen doses and using two distinct adjuvants
Source: BMC Immunol. 2017 Jul 26;18:39. doi: 10.1186/s12865-017-0222-z (PMC5530503; doi:10.1186/s12865-017-0222-z)
Supplement: Supplementary file 3 — Detection of specific-IgG, IgM or IgA immunoglobulin classes at the end of trial vaccinations (week 13). (PDF 100 kb) [file 12865_2017_222_MOESM3_ESM.pdf]

**Additional file 3.** Detection of specific-IgG, IgM or IgA immunoglobulin classes at the end of trial vaccinations (week 13).

| | Patients | IgG | IgM | IgA |
|---|---|---|---|---|
| Group Ag+V | CQ03 | + | - | - |
| | CH04 | - | - | - |
| | CH17 | + | - | - |
| | CH24 | + | - | - |
| | CH32 | + | - | + |
| | JL41 | + | + | + |
| | JL42 | + | - | - |
| Group Ag+2V | CH15 | - | - | - |
| | CH19 | + | + | + |
| | CH27 | + | - | - |
| | CH39 | - | - | - |
| | JL49 | + | + | - |
| | JL22 | - | - | - |
| | CH06 | + | - | + |
| | CQ44 | - | - | - |
| Group 2Ag+V | CH16 | + | + | + |
| | JL29 | + | - | + |
| | JL30 | + | - | - |
| | CH33 | + | + | + |
| | JL43 | + | - | - |
| | CH50 | + | - | + |
| | JL12 | + | + | - |
| | CH35 | + | - | + |
| Group ½Ag+Al | CH08 | - | - | - |
| | CH09 | - | - | - |
| | CH20 | - | - | - |
| | CH25 | - | - | - |
| | CH46 | - | - | - |
| | JL47 | - | - | - |
| | CH18 | - | - | - |
| | JL23 | + | - | - |
| Group Ag+Al | CH07 | + | - | - |
| | CQ13 | + | - | - |
| | CQ28 | + | - | - |
| | CH37 | + | - | - |
| | CH45 | + | + | + |
| | JL48 | + | - | + |
| | JL11 | - | - | - |
| | CH10 | + | - | - |

(+): sample meets acceptance criteria for antibody titer; (-): sample does not meet acceptance criteria for antibody titer.
