# Supplementary material for: Characteristics of the specific humoral response in patients with advanced solid tumors after active immunotherapy with a VEGF vaccine, at different antigen doses and using two distinct adjuvants
Source: BMC Immunol. 2017 Jul 26;18:39. doi: 10.1186/s12865-017-0222-z (PMC5530503; doi:10.1186/s12865-017-0222-z)
Supplement: Supplementary file 5 — Off-trial re-immunizations. (PDF 97 kb) [file 12865_2017_222_MOESM5_ESM.pdf]

Additional file 5. Off-trial re-immunizations.

Patient Code	Number of re-immunizations	Sample evaluations (weeks)	Time of dose change (week)
**Vaccination dose: 400µg antigen+200µg VSSP**			
CQ03	7	(16)	No
CH04	3	(16)	No
CH17	5	(16)	No
CH24	4	(16,24)	No
CH32	6	(16,24,32)	No
JL41	5	(16,18,28)	No
JL42	10	(16,18,28,36,44,49,50)	No
**From initial vaccination dose [400µg antigen+400µg VSSP] to 800µg antigen+200µg VSSP**			
CH15	1	(16)	No
CH19	9	(16,32,40,47,48)	(36)
CH27	9	(16,32,45,46)	(36)
CH39	4	(16,24,28)	(28)
JL49	9	(16,24,30,38,44,45)	(28)
**Vaccination dose: 800µg antigen+200µg VSSP**			
CH16	8	(16,32)	No
JL29	10	(16,32,40,48,49)	No
JL30	11	(16,34,42,50,52,53,60)	No
CH33	9	(16,24,32,40,48,49)	No
JL43	5	(16,18,28)	No
CH50	2	(16)	No
JL12	9	(16,20,40,53,54)	No
**From initial antigen dose [200µg antigen+0.7mg Al^{3+}] to 800µg antigen+200µg VSSP**			
CH08	9	(16,55,56)	(44)
CH09	1	(16)	No
CH20	9	(16,36,46,50,51)	(40)
CH25	1	(16)	No
CH46	4	(16,24,36)	(28)
JL47	1	(16)	No
**From initial antigen dose [400µg antigen+0.7mg Al^{3+}] to 800µg antigen+200µg VSSP**			
CH07	9	(16,40,48)	(40)
CQ13	9	(16,36,48,49)	(44)
CQ28	1	(16,30)	No
CH37	1	(16)	No
CH45	8	(16,24,28,32,40,48,49)	(36)
JL48	4	(16,24)	(28)
JL11	1	(16)	No
